# The Effect of COVID-19-related Lockdowns on Diet and Physical Activity in Older Adults: A Systematic Review

**DOI:** 10.14336/AD.2021.0606

**Published:** 2021-12-01

**Authors:** Elisabeth Anne Larson, Karlen Stade Bader-Larsen, Faidon Magkos

**Affiliations:** Department of Nutrition, Exercise and Sports, University of Copenhagen, Frederiksberg C, Denmark

**Keywords:** nutrition, exercise, sedentary, coronavirus, elderly

ABSTRACT: The lockdown restrictions imposed globally to curb the COVID-19 pandemic have altered many aspects of daily life, including diet and physical activity. The aim of this systematic review was to evaluate evidence for changes in the diet and physical activity habits of older adults due to COVID-19-related lockdowns. We included analytic observational studies that reported on changes in diet, physical activity, or both, among older individuals (≥50 years old). We searched PubMed and EBSCO LISTA to identify original research articles published between 01/2020-03/2021. We identified 27 studies, 5 of which reported on changes in diet, 17 on changes in physical activity, and 5 on changes in both. The sample sizes ranged from 17 to 3110 subjects. Six of 10 papers on diet reported no significant changes in quantity or quality of food consumption; of those who did find changes in diet, these were generally unfavorable. Thirteen of 22 studies on physical activity reported a decrease in physical activity or an increase in sedentary time; the rest reported no major changes. Pre-lockdown habits were a predictor of change in some studies. The safer-at-home measures have not greatly impacted the diet of older adults but have led to a significant decrease in their physical activity, putting them at higher risk for non-communicable diseases, which may further increase their susceptibility to COVID-19. Ultimately, these findings may help guide clinical practice, by promoting additional health screenings by general practitioners for the elderly and by emphasizing the need for lifestyle interventions like at-home exercise initiatives, to help mitigate the negative impact of the pandemic on this especially vulnerable age group.

## Introduction

On December 31, 2019, the World Health Organization (WHO) Office in the People’s Republic of China received a media statement from the Wuhan Municipal Health Commission regarding a ‘viral pneumonia’ spreading in Wuhan, China (www.who.int/emergencies/diseases/novel-coronavirus-2019/interactive-timeline) [1]. The pneumonia, since named coronavirus disease 2019 (COVID-19), proliferated rapidly, with cases appearing globally by the end of January 2020. As a result, COVID-19 was quickly labeled a Public Health Emergency of International Concern by the WHO (www.who.int/emergencies/diseases/novel-coronavirus-2019/interactive-timeline). Evidence for human-to-human transmission mounted as infections were identified in healthcare workers caring for infected patients, and in February 2020, the WHO released global guidelines for quarantine of infected individuals [1] (https://www.who.int/emergencies/diseases/novel-coronavirus-2019/interactive-timeline). In March 2020, many countries enacted social distancing or lockdown orders, aimed at curbing the spread of the disease. These orders varied in their severity but included closures of schools and non-essential businesses, work-from-home recommendations, bans on public gatherings and even home isolation orders (www.who.int/emergencies/diseases/novel-coronavirus-2019/interactive-timeline). Still, by April 19, 2021, there were over 140 million confirmed cases of COVID-19 worldwide and more than 3 million deaths (https://covid19.who.int).

These safer-at-home orders gave rise to a public health conundrum. Home confinement could lead to reduced physical activity, increased sedentary time, and unfavorable changes in dietary habits. In turn, these changes could result in weight gain and a greater risk for non-communicable diseases [2, 3] which, paradoxically, increase susceptibility to COVID-19 (https://www.cdc.gov/coronavirus/2019-ncov/need-extra-precautions/people-with-medical-conditions.html, https://sdg.iisd.org/news/who-finds-non-communicable-diseases-heighten-risk-of-severe-covid-19). Pandemic-related lockdown measures could impact the quantity and quality of the diet in several ways. Limiting grocery trips may alter the types and amounts of foods purchased, causing a shift from fresh foods to more shelf stable products with poorer nutritional value; pandemic-related anxiety may increase emotional eating; and more time at home could give rise to more snacking opportunities. However, with restaurants closed, there could be more time to prepare healthier, home-cooked meals [4]. Regarding physical activity, between April and May 2020, 41 countries issued stay-at-home orders, with the most severe resulting in total home confinement [5]. This restricted access to gyms, outdoor fitness infrastructures, and ultimately to previously typical modes of exercise, making it more difficult for individuals to maintain their earlier patterns of physical activity.

Older age is associated with a greater risk for a severe course of illness with COVID-19 due to age-related changes in physiological functions and increased comorbidities [6]. For several reasons, older individuals are also particularly vulnerable to the downstream effects of the COVID-19 lockdowns. Social isolation, physical disability, and socioeconomic hardship can contribute to poor exercise habits and limit access to healthy food for older adults, even prior to COVID-19 [7]. With the added burden of pandemic-related lockdowns, more time spent indoors may impact their ability to perform social physical activities they may have previously been engaged in [8], and fear of illness may result in fewer outings, thus affecting their grocery shopping patterns and eating habits even further.

To fully comprehend the impact of the COVID-19 pandemic, the public health implications of containment efforts must also be understood. The aim of this systematic review was to conduct a thorough analysis of the current literature on the effects of the COVID-19-related lockdowns on diet and physical activity habits in older individuals.

## METHODS

This systematic review was conducted following the Preferred Reporting Items for Systematic Review and Meta-Analysis checklist [9], and the protocol has been registered with the PROSPERO database (registration number: CRD42021241997).

### Search strategy

A comprehensive, systematic database search was conducted in March 2021, using the databases PubMed and EBSCO LISTA. The following keywords and index terms were used: “nutrition” or “food” or “diet” or “exercise” or “physical activity” AND “COVID-19” or “coronavirus” or “Sars-CoV-2” or “acute respiratory syndrome” AND “lockdown” or “restrictions” or “curfew” or “quarantine” or “shutdown.” The search was restricted to the time period of January 2020 - March 2021, but had no restrictions on language, study type, or place of publication.

### Eligibility criteria

We included original research articles assessing changes in diet, physical activity, or both, as a result of COVID-19-related lockdowns. Publications must have reported on the general adult population or specifically among older adults.

Additionally, we excluded studies that: 1) did not pertain directly to our research question 2) were conducted in specific subgroups of the population such as pregnant women, athletes, and groups of individuals with specific pathological diagnoses; 3) were conducted in infants, children or adolescents; 4) were not published in the English language; 5) were case reports, letters to the editor, recommendations, or interventional studies; 6) did not provide pre-lockdown data (i.e. “baseline” data for the period before the lockdown measures commenced), acquired either prospectively or retrospectively; and 7) did not report results by age group and specifically for the older age group(s). We defined older adults as those 50 years of age or older after reviewing the age groupings most often used in the retrieved primary studies, as well as those used in previous systematic reviews [8].

### Data extraction

Two members of the research team (EAL, KSBL) created and refined a tool to extract data for each of the publications included in the review. The following information was retrieved: first author, study population and location, period of study, period and specifics of the lockdown (if available), methods (including type and format of questionnaires used), outcome measures for total population, and older age group-specific results.

### Risk of bias assessment

The ROBINS-I tool for non-randomized studies [10], adapted for analytic observational studies that have measurements before and after an intervention or exposure and lack a control group [11], was used to assess risk of bias. The following domains were evaluated: selection of participants to the study, missing data, measurement of exposure, measurement of outcome, selection of reported result, and confounding.

## RESULTS

**Figure 1.**
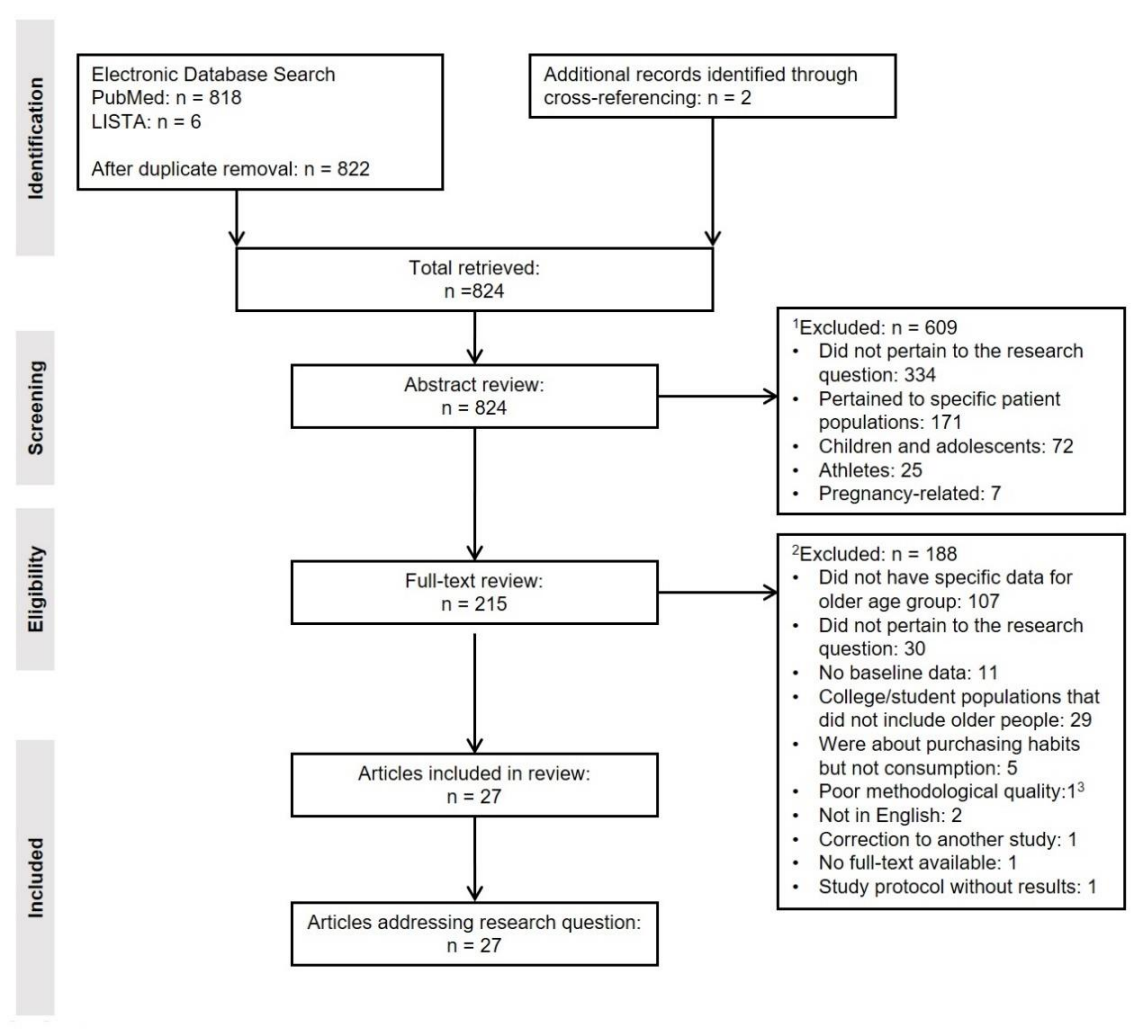
**PRISMA Flow Diagram of article search and selection process.** ^1^Abstract did not meet the following inclusion criteria: i) adult population (ages 18+), ii) addressed changes in diet and/or physical activity due to restrictions and social distancing measures implemented as a result of the COVID-19 pandemic, iii) members of the general population (i.e., excluded athletes, those in a cohort carrying a specific disease, those with specific mental health diagnoses, those living in nursing homes, etc.). ^2^Article did not meet the following inclusion criteria: i) change in diet and/or PA in older population (ages 50+), ii) pre-lockdown data, iii) methods considered sound by the research team, and iv) analytic observational studies (i.e., excluded letters to the editor, case studies with n=1, etc.). ^3^Poor Methodological Quality: Qualitative study with leading questions

A total of 822 unique articles were identified from the database search, and two more were found through cross-referencing, for a total of 824 articles; the PRISMA flow diagram is shown in Figure 1. The titles and abstracts of these articles were reviewed by the research team, resulting in 215 eligible articles for further evaluation. Two members of the research team (EAL, KSBL) reviewed the full text of each publication and met to discuss any discrepancies to reach joint consensus. The third member (FM) resolved any disagreements. This process resulted in a final 27 articles meeting the inclusion criteria (Fig. 1).

**Table 1:** Characteristics of the included studies.

Author	Population	Study period / Lockdown details	Methods	Outcomes	Results
**Alhusseni and Alqahtani [12]**	• Saudi Arabian adults • Total n=2706 • Older (age >55 years): n=149	• Study from May 5 to 15, 2020 • Lockdown from March to June, 2020 • Restaurants, shops and outdoor sports facilities closed. Only online food purchases	• Online survey • Retrospective self-report for baseline data • Used Likert scale and food frequency scale	• Δ^1^ in diet quality and quantity	• Food quantity ? but quality ? in the 55+ age group during the lockdown period
**Belgen Kaygisiz et al. [13]**	• Northern Cypriot females • n=104 • Mean age ~59?years	• Study from April 23 to 27, 2020 • Lockdown not described	• Online survey using Beck anxiety questionnaire, IPAQ-SF^2^ • Levels of PA^3^ in previous week • IPAQ^4^ score converted to level of activity • Retrospective self-report for baseline data	• Δ in PA and anxiety	• Those with regular PA at baseline had higher lockdown PA than those who were not regular exercisers • 52% had regular exercise pre-pandemic and 30% did during • ? anxiety associated with ? PA
**Bourdas and Zacharakis [14]**	• Greek adults • Total n=8495 • Older (age 50-70+): n=2008	• Study from April 4 to 19, 2020 • Lockdown from March 23 to May 4, 2020 • Leaving home allowed only for commuting, essential errands, and exercising	• Online questionnaire “Active-Q” for PA • Frequency and duration of PA across 4 domains • Converted to MET^5^-min/week • Retrospective self-report for baseline data	• Δ in PA	• 50-59 age group, 15% ? PA • 60-69 age group, 19% ? PA • 70+ age group, 32% ? PA
**Brown et al. [15]**	• Older UK adults • n=142 • Age 76-97 years	• Study from May 14 to June 1, 2020 • Lockdown included ban of nonessential travel; no contact with individuals outside the home	• Telephone survey • Participants from Community Ageing Research 75+ cohort study • Retrospective self-report for baseline data	• Δ in PA	• 42% ? PA • 17% ? PA • 60% did PA everyday
**Cancello et al. [16]**	• Northern Italian adults • Total n=490 • Older (age >60 years): n=100	• Study from April 5 to May 4, 2020 • Lockdown included individuals could leave home only for urgent needs, most working from home	• Online survey • Questions regarding hours of PA, food intake changes • Retrospective self-report for baseline data	• Δ in PA	• 60+ age group had a decreased chance of ? PA (OR^6^ 0.21) and an increased chance of ? PA (OR 1.22) compared to those ≤30 years
**Castaneda-Babarro et al. [17]**	• Spanish adults • Total n=3800 • Older (age 55-65 years): n=347	• Study from March 23 to April 1, 2020 • No details on lockdown measures	• Online survey • IPAQ short version • Questions about leisure time PA in 4 domains • Retrospective self-report for baseline data	• Δ in time and intensity of PA • Δ in sitting time	• Vigorous PA ? by 44 min/week • Walking time ? 194 min/week • No significant increase in sitting time
**Cicero et al. [18]**	Italian older adults • n=359 • Mean age 64.6 years	• Study from February to April 2020 • Lockdown described as “strict quarantine”	• Phone interviews using DQI^7^ • Validated tool for usual food intake of 18 food items • Retrospective self-report for baseline data	• Δ in dietary habits	• 19% began taking dietary supplements • Majority (50%) did not change diet • In those who did change, ? carbs and fats and alcohol
**Constandt et al. [19]**	• Belgian adults • Total n=13515 • Older (age 55-74 years): n=4739	• Study from March 30 to April 5, 2020 • Lockdown from March 13 to May 4, 2020 • Going to work and outdoor exercise permitted, schools closed	• Online survey • Retrospective self-report for baseline data • Separate analyses for high and low active groups	• Δ in amount and type of PA	• Ages 55-74 more likely to fall in a lower PA category during the lockdown, compared with younger groups
**Constant et al. [20]**	• French adults • Total n=4005 • Older (age 60+ years): n =1032	• Study from April 8 to 20, 2020 • Lockdown: home confinement with allotment for daily exercise	• Online survey • 5 health behaviors (screen watching, snacking, eating fruits and vegetables, exercising, walking) frequency pre and post • Retrospective self-report for baseline data	• Δ in lifestyle including eating behaviors	• Healthy changes in eating habits were negatively related to being aged 40 years or older • Higher levels of alcohol drinking decreased with age
**Deschasaux-Tanguy et al. [21]**	• French adults • Total n=37252 • Older (age 50-65, 65-80, 80+): n not reported	• Study from April to May 2020 • Lockdown from March 17 to June 2, 2020 • Closing of nonessential business, stay-at-home order, leaving the house only for essential shopping	• Online questionnaire • IPAQ for PA, 24-hr diet records for dietary intake • Baseline data from last administration of questionnaires (NutriNet-Santé cohort) • Self-report for post-lockdown changes	• Δ in PA • Δ in dietary intake	• Those older than 50 years were most likely to fall into the cluster that experienced no change in diet or PA since the start of the pandemic
**Eek et al. [22]**	• Swedish adults • Total n=1318 • Older (age 70+ years): n=61	• Study from September 1 to 7, 2020 • Online school, work from home, restricted activities for fitness centers, gathering restrictions	• Online questionnaire • IPAQ-SF for frequency and duration of PA • Step counts for those with smart devices • Retrospective self-report for baseline data	• Δ in extent and intensity of PA	• The highest odds of ? PA was found in those aged 70+ years (OR 2.8) • 55% of those aged 70+ ? PA, as well as 30% of those aged 50-69
**Faulkner et al. [23]**	• UK, Ireland, New Zealand and Australia • Total n=8425 • Older (age 50+ years): n=3110	• Study from April to May 2020 • Type of lockdown not specified	• Online survey • Stage of Change for exercise behavior • IPAQ-SF • Retrospective self-report for baseline data	• Δ in PA (positive or negative)	• Majority reported no change in PA habits and behavior
**Ferrante et al. [24]**	• Italian adults • Total n=7847 • Older (age 50-69 years): n=3521; (age 70+ years): n=446	• Study from April to June 2020 • Lockdown details not specified	• Online questionnaire • Subsections about demographics, leisure time PA, substance use, eating habits, health status and mental health • Retrospective self-report for baseline data	• Δ in PA • Δ in food habits	• ? PA of 62% and 57% in those aged 50-69 years and 70+ years •No significant change in alcohol for those aged 50-69 •5% ? in alcohol in those aged 70+ •No significant change in other food habits
**Franco et al. [25]**	• Southern Italian adults • Total n=310 • Older (age 65+ years): n=17	• Study from May 12 to June 12, 2020 • Lockdown included travel restrictions, close of nonessential businesses, stay-at-home advice	• Online Survey • IPAQ-SF, adapted • PA levels in MET-min/week • Retrospective self-report for baseline data	• Δ in PA-related energy expenditure	• ? 20 MET-min/week among the older group (aged 65+ years)
**Giebel et al. [26]**	• Older Ugandan adults • n=30 • Age 60+ years	• Study in June 2020 • Lockdown included social distancing and curfew	• Semi-structured phone interviews • Questions about changes to daily life • Retrospective self-report for baseline data	• Δ in food consumption patterns	• ? number of meals with consistent hunger, because of being unable to receive gov’t meals, or dislike of gov’t meals, or lack of access to normal farming
**Lamarche et al. [27]**	• Canadian adults • Total n=853 • Older (age 50+ years): n=538	• Study from April 15 to May 12, 2020 • No lockdown details	• Online questionnaire • 24-hr recall, HEI^8^ for diet quality, food security questions, PA questionnaire • Baseline data collected upon recruitment (June 2019) at the NutriQuébec cohort	• Δ in HEI	• Healthy eating ? in those aged 50-69 years but ? slightly in those aged 70+ years
**Okely et al. [28]**	• Scottish adults • n=137 • Mean age 84 years	• Study on 27 May 2020 (34 days after start of national lockdown) • Lockdown details not provided	• Sent paper questionnaires by mail • One item for PA (usual level of PA) • Answered with Likert scale • Asked to respond to questions twice, once for pre-lockdown, and once for post • Retrospective self-report for baseline data	• Δ in PA level	• Overall ? in PA (P=0.012) • ? in low-intensity PA (only performing chores), and ? in moderate/high-intensity PA
**Perez-Rodrigo et al. [29]**	• Spanish adults • Total n=1155 • Older (age > 55 years): n=379	• Study from April 21 to May 8, 2020 • Lockdown from March 15 to May 2, 2020. • Only essential services open; could leave home to buy food but not for exercise	• Online Questionnaire using FFQ^9^ (validated) • FFQ data used to compute score that rated adherence to Dietary Guidelines of SENC^10^ • 2^nd^ food section about changes in consumption during confinement (not validated) • Questions about time and intensity of PA as well as sedentary time • Retrospective self-report for baseline data	• Δ in PA patterns • Δ in dietary habits	• People aged >55 years had ? odds of being in more physically active cluster compared to those aged <55 years
**Richardson et al. [30]**	• Older UK adults • n=117 • Age 70+ years	• Study from March 11 to May 4, 2020 • Lockdown started on March 20, 2020 • Included closures of gyms, restaurants, and social venues; stay-at-home advice	• Online Survey • First administered March 11 to 28 for baseline data (retrospective for some) • Additional survey administration Q14 days from March 1 to May 4, 2020 • IPAQ, questions with frequency, duration, and intensity of PA • IPAQ answers converted to MET-min/week	• Δ in PA level	• PA in MET-min did not ? • Time spent sitting ? • 79% changed habitual activities to stay active
**Rolland et al. [31]**	• French adults • Total n=11391 • Older (50-64 years): n=2043; (65-74 years): n=547; (75+ years): n=81	• Study from March 24 to 30, 2020 • Lockdown started on March 16, 2020 • Only essential activities allowed, related to medical care and to food supply	• Online Questionnaire WEMWBS^11^ • Section F about Δ in intake of coffee/tea/energy drinks, and caloric/fatty/sweet/salty foods, and alcohol • Retrospective self-report for baseline data	• Δ in calorie-rich and salt-rich food intake	• In those aged 50-64 years, 50% did not increase their caloric/salty foods. • The same was observed in those aged 65+ years
**Rossinot et al. [32]**	• French adults • Total n=1454 • Older (age 55-64 years): n=225	• Study from April to May 2020 • Lockdown from March 17 to May 11, 2020 • Travel only for food shopping, medical appointments, work; limited outdoor outings for exercise; threat of fine or imprisonment for noncompliance	• Online questionnaire (researcher's own) • Retrospective self-report for baseline data • Questions about change in “balanced-ness” of diet, sleep, physical activity, tensions with relatives, tobacco and alcohol consumption	• Δ in diet • Δ in PA • Δ in alcohol consumption	• No statistically significant changes in nutrition, alcohol consumption or PA
**Sasaki et al. [33]**	• Older Japanese adults • n=999 • Age 65-90 years	• Study in August 2020 • No data on lockdown measures	• IPAQ-SF by mail • Frequency and duration of MVPA^12^ • Retrospective self-report for baseline data (asked to report on October 2019 habits) •Responses converted to MET-min/week • MET classified into low, medium or high based on national PA guidelines	• Δ in amount and type of PA	• Total PA ? 5-10% • ? in sitting time (5% for males, 10% for females) • Males ? high-intensity PA (but not the females) • Females with a low socioeconomic status did not ? PA • Women with social participation ? or maintained MVPA • Roughly half of females and males ? MVPA but maintained low-intensity PA while the other half ? overall PA or maintained MVPA
**Schlichtiger et al. [34]**	• Bavarian older adults • n=110 • Age 50+ years	• Survey from March to April 2020 • Lockdown details included curfew with exceptions for work, necessary shopping, medical visits, assisting others, visits from partners, and exercise	• Online survey based on the validated PA Questionnaire 50+ • Adapted to include questions for baseline demographic and health data • Converted answers to MET-hours/week • Retrospective self-report for baseline data	• Δ in PA level	• ? in PA (MET-hours/week) and energy expenditure (kcal/week) (both P<0.001) • Median ? from 162 to 140 MET-hours/week •Leisure activities, sports, and work ? • ? in yardwork
**Suzuki et al. [35]**	• Japanese older adults • n=165 • Age 65+ years (mean age 79 years)	• Study from March 20 to May 13, 2020 • Lockdown included schools, recreational and commercial facilities closed, with stay-at-home orders in place	• Questionnaire mailed to participants in two waves • Wave 1: retrospective baseline data at start of pandemic • Wave 2: behavior changes •PAQ-EJ^13^ score converted to MET-hours/week • PA with 7 subscales with different types of PA activity • Questions added about change in PA	• Δ in amount, type and frequency of PA	• Those who were less active at baseline: 38% ? in PA across all categories • Those who were more active at baseline: 47% ? in PA across all categories
**Visser et al. [36]**	• Dutch older adults • n=1119 • Age 62-98 years	• Study from June 8 to October 8, 2020 • No lockdown details	• Questionnaire, via phone, paper or online • Retrospective self-report for change in 7 nutrition behaviors and change in PA • PA LAPAQ^14^ and validated with pedometer counts and activity diaries	• Δ in eating behavior • Δ in PA	• 87% reported not eating less than normal • 84% reported never drinking more alcoholic beverages • 64% reported not gaining weight • Most reported getting enough PA to meet recommendations
**Werneck et al. [37]**	• Brazilian adults • n=38353 • Older (age 60+ years): n not reported	• Study from May 24 to April 24, 2020 • No lockdown details	• Online survey about PA and TV viewing • Activities classified based on movement recommendation of 150 min/week, and electronic use (cutoff of 4+ hours/day defined as “high use”) • Retrospective self-report for baseline data	• Δ in frequency and duration of PA • Δ in TV and PC use	• Those who were inactive with ? TV use: ? prevalence of unhealthy movement behaviors by 3% • Those who were inactive with ? PC use: ? prevalence of unhealthy movement behaviors by 7% • Those who were inactive with ? TV and ? PC use: ? prevalence of unhealthy movement behaviors by 16.7%
**Williams et al. [38]**	• Scottish adults • Total n=3342 • Older (age 50-64 years): n=1130; (age 65+ years): n=402	• Study from May 20 to June 12, 2020 • Lockdown started on March 2020 • Stay-at-home order, could leave only for essentials like food shopping or medical purposes or exercise	• Online Qualtrics Survey • Questions about demographics, health, and “positive changes” • Positive changes of PA, diet, and other measures of daily life measured with positive events subscale of the EPII^15^ • Results presented as cumulative change, no subsection for diet and PA • High percent positive = more positive change • Retrospective self-report for baseline data	• Δ in “positive behaviors”	• Those aged 65+ years demonstrated the lowest level of positive change • Those aged 65+ years had a positive change score 8% lower than youngest age group

Abbreviations used:

1 Δ, change;

2 IPAQ-SF, International Physical Activity Questionnaire-Short Form;

3 PA, Physical Activity;

4 IPAQ, International Physical Activity Questionnaire;

5 MET, Metabolic Equivalent of Task;

6 OR, Odds ratio;

7 DQI, Dietary Quality Index;

8 HEI, Healthy Eating Index;

9 FFQ, Food Frequency Questionnaire;

10 SENC, Spanish Society of Community Nutrition;

11 WEMWBS, Warwick-Edinburgh Mental Well-Being Scale;

12 MVPA, Moderate-Vigorous Physical Activity;

13 PAQ-EJ, Physical Activity Questionnaire-Elderly Japanese;

14 LAPAQ, Longitudinal Aging Study Amsterdam Physical Activity Questionnaire;

15 EPII, Epidemic-Pandemic Impacts Inventory.

### Characteristics of the included studies

The methodological characteristics and key findings of the 27 articles included in this review are provided in Table 1 [12-38].

Five studies exclusively reported on changes in dietary behaviors in older individuals [12, 18, 26, 27, 31], 17 exclusively reported on changes in physical activity [13-17, 19, 22, 23, 25, 28-30, 33-35, 37, 38], and 5 reported on both [20, 21, 24, 32, 36]. Many (n = 17) studies reported on a variety of diet and/or physical activity outcomes for the total study population but provided only selective results for the specified age group of 50+ years [12, 14, 16, 17, 19-25, 27, 29, 31, 32, 37, 38]. With the exception of 2 articles [21, 27] that collected data concurrently as part of an independent cohort study before COVID-19-related lockdowns, the majority (n = 25) relied on retrospective self-report for pre-lockdown data. The sample sizes of older age groups ranged from 17 to 3110 individuals, included both males and females (1 study reporting only in females), from 6 continents, with the majority of the studies coming from European researchers and locations (Table 1).

Lockdown information was not reported in all studies. In those that did report lockdown details, the restrictions ranged from assembly bans and limits with curfews and social distancing guidelines to near complete home quarantines, with individuals only allowed outdoors for purchasing food and urgent medical appointments.

**Figure 2.**
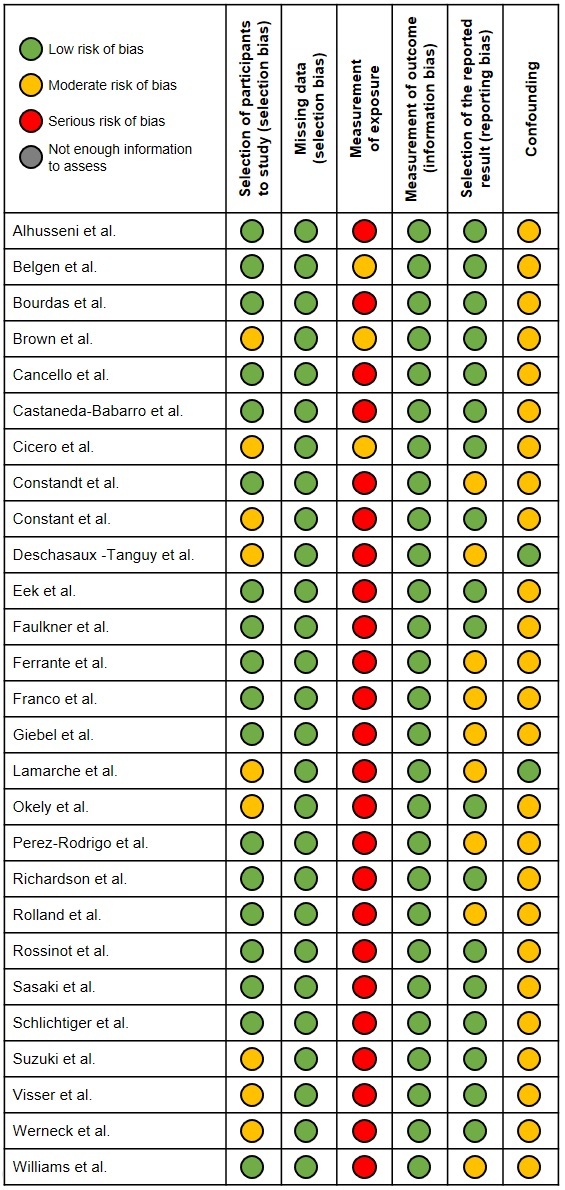
Risk of bias assessment of included studies (ROBINS-I tool).

**Figure 3.**
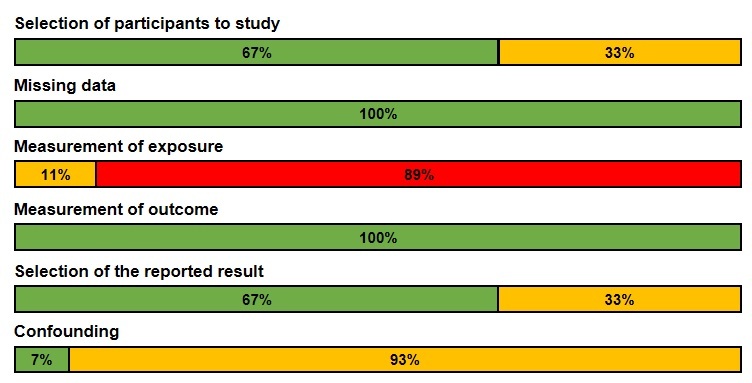
Distribution of included studies according to bias.

All of the studies contained some bias, to varying degrees of severity (Fig. 2 and 3). Most (89%) had severe bias in measurement of the exposure as the researchers did not retrieve data from participants about the level of social-distancing measures they had undertaken. A few (11%) had home quarantine as an inclusion criterion or used questionnaires that probed into the extent of the participants’ social distancing practices. Most of the studies (93%) also had moderate confounding caused by insufficient timepoints by which to extrapolate trends. There was moderate bias in selection of reported results (33%) as several studies did not report results for all outcomes in all age groups. These biases should be considered when interpreting the results, as they negatively impact the robustness of the findings.

### Changes in diet

Ten articles addressed the impact of the COVID-19-related lockdowns on diet [12, 18, 20, 21, 24, 26, 27, 31, 32, 36] and 6 observed no significant changes in the dietary habits of older adults [18, 21, 24, 31, 32, 36].

Cicero et al. [18] used the Dietary Quality Index, a validated questionnaire that assesses typical intake of 18 food items, and found that 50% of respondents did not alter their diet quality during the lockdown period. Similarly, Deschaseux et al. [21] reported that those over the age of 50 years were more likely to fall in the cluster that reported no change in dietary habits after the start of the pandemic (ages 50-65 years, OR=1.40; ages 65-80 years, OR=2.02; ages 80+ years, OR=2.10; compared to the reference group of ages 25-50 years). These findings were echoed by Visser et al. [36], who reported that 87% of subjects did not eat less than normal (p=0.003), and that 64% did not gain weight since the start of the pandemic, indicating a relative maintenance of pre-lockdown behaviors. Rolland et al. [31] found that among those 50 years and older, 50% did not increase their intake of calorie-rich or salt-rich foods; and Rossinot et al. [32] found no significant changes in diet or alcohol consumption.

The overall trend across the 10 studies that evaluated dietary habits during the lockdown was that of no change from before lockdown, but several studies presented a different narrative that was not always consistent. A qualitative study out of Uganda by Giebel et al. [26] reported that many of the older participants consumed fewer daily meals, and had a concurrent increase in hunger. In contrast to that, Alhusseini and Alqahtani [12] reported that the older Saudi Arabian adults in their study increased food quantity (p=0.003) but also decreased food quality (p=0.01). Differences in the socioeconomic status and food availability between these 2 countries may be partly responsible for these diametrically opposite results. Constant et al. [20] found that individuals aged 40-60 and 60+ years were ~20% less likely to have made a “positive” lifestyle change in eating habits during the lockdown period in France. On the other hand, in Canada, Lamarche et al. [27] reported an increase in healthy eating behaviors among those aged 50-69 years (p=0.02), but a slight decrease in healthy behaviors among those aged 70+ years (p<0.001).

### Changes in physical activity

Twenty-two articles reported on changes in physical activity as a result of the COVID-19 lockdown measures [13-17, 19-25, 28-30, 32-38]. In contrast to the results for diet, most of this literature reported a decrease in physical activity and/or an increase in sedentary time in the older population [13-17, 19, 22, 24, 25, 28-30, 33, 34, 37, 38].

To highlight this, a study in older Spanish adults from Castaneda-Barbarro et al. [17] reported an average decrease of 44 minutes per week in vigorous physical activity (p<0.001), and an even larger decrease in walking activity of 194 minutes per week (p<0.001). Sasaki et al. [33] mirrored this finding, reporting a 5-10% decrease in overall physical activity in older Japanese adults, with a similar percent increase in time spent sitting. Richardson et al. [30] also observed an increase in time spent sitting, and Okely et al. [28] found an overall decline in physical activity, with an increased proportion of participants falling into a low physical activity category (only performing household chores) and a lower proportion maintaining heavy physical activity (p=0.012) during the lockdown when compared to pre-restriction habits.

The results for change in physical activity habits were not homogenous, however, and some studies found evidence that pre-pandemic habits may have impacted lockdown-induced changes. For example, Suzuki et al. [35] reported a 38% decline across all types of physical activity for those who were less active at baseline (p<0.01) but a 47% increase across all types of physical activity among those who reported high levels of physical activity at baseline (p<0.01). Similarly, Werneck et al. [37] found that those who were inactive with high PC and TV use before the pandemic increased their unhealthy movement behaviors by 17% during the lockdown. Social engagement was a protective factor for maintaining physical activity among older adults [33], while anxiety was detrimental towards overall physical activity levels [13].

## DISCUSSION

This review synthesized the currently available evidence regarding the effects of the COVID-19-related lockdowns on diet and physical activity in older adults. Our findings suggest that diet was largely unaffected by pandemic-imposed lockdowns, although the majority of the studies identified were conducted in high-income countries. These results were contrasted starkly with 1 study in a low-income setting, where food consumption decreased [26]. For the small number of studies that did report changes in diet, the changes were generally unfavorable, involving either increased food consumption [12] or increased quantity of calorie-rich and nutrient-poor foods [18]. For physical activity, the general trend for older adults showed a decrease, with pre-pandemic exercise and social engagement as predictors of maintenance of physical activity levels [33, 35]. Increased sitting or screen time tended to correlate with decreased overall physical activity levels [37].

Fourteen months after the WHO first deemed COVID-19 a public health emergency, daily life remains disrupted worldwide (www.who.int/emergencies/diseases/novel-coronavirus-2019/interactive-timeline).

This review highlights the need for a greater focus on the particularly vulnerable older adults. While the available studies contribute much needed knowledge about the effects of the COVID-19-related lockdowns on the general population, they also reveal a significant gap in the evidence base regarding specific effects on this particularly susceptible age group. The older age demographic, whether as the study focus or as a subcategory, was grossly underrepresented. Our results suggest that old age alone was not a risk factor for unfavorable diet changes [18, 21, 24, 31, 32, 36]. More concerning, though, is the general trend towards reduced physical activity [13-17, 19, 22, 24, 25, 28-30, 33, 34, 37, 38]. The importance of physical activity in the older population is well established (www.cdc.gov/physicalactivity/basics/older_adults/index.htm). Strength training, paired with aerobic exercise and balance work, is crucial to preventing falls and fractures in older individuals [39]. Lack of such physical activity can drive body composition changes, with declines in lean body mass and increased risk of developing sarcopenia. Additionally, declines in physical activity put the elderly at risk for cardiovascular events and other non-communicable diseases [39]. Reduction in total energy expenditure through a decrease in physical activity and exercise could have detrimental effects on body weight [2, 3], and thereby also on metabolic homeostasis. However, no studies collected biological material or conducted relevant tests, and the few that collected self-reported weight and height data did not specifically report those results for the older age groups.

Reduction in physical activity as a result of the pandemic is concerning, particularly among older individuals for whom access to outdoors facilities (e.g. parks or other green areas, public fitness corners) is important for maintaining a physically active lifestyle [40]. Already at risk of adverse events related to aging, older individuals now face the added challenge of maintaining their health amidst the restrictions imposed as a result of the pandemic. General practitioners and public health officials may need to focus on encouraging physical activity in this age group to decrease risk of cardiovascular disease and improve cardiometabolic outcomes. This will require not only motivating older individuals to overcome a number of existing challenges (e.g. accessibility, illnesses, lack of a peer group, a sense of disempowerment, and fear of injury) [41]; but also some innovative solutions under the current circumstances, possibly involving household chores, gardening, online group activities, or focusing on decreasing sedentary activities. Home-based exercise, for instance, appears effective to improve several components of physical fitness and health in older adults (i.e. muscular strength, endurance, power, and balance) [42]. Attention to our results can fuel data-driven public health interventions aimed at mitigating the effects of the COVID-19-related lockdowns on the lifestyle and health of older individuals.

### Strengths and limitations

Our systematic review has several limitations, many of which relate to the challenge of conducting research in a pandemic [4, 43]. The pandemic itself imposed many restrictions on researchers and their respective studies included in this review. The speed at which countries reacted to the emerging pandemic and responded with social distancing measures or lockdowns prevented researchers from collecting baseline data in real time. As such, the pre-restriction data provided was likely subject to considerable reporting bias from the participants as they were asked to recall previous behaviors that they may not accurately remember [44-46].

Additionally, we were challenged by the heterogeneous methodologies employed to assess diet and physical activity across studies, with only a few researchers utilizing validated tools (Table 1). For instance, although some authors used the International Physical Activity Questionnaire (IPAQ) or the Diet Quality Index (DQI), many ended up creating their own questionnaires that specifically pertained to pandemic-related changes, but without proper validation. We were also surprised that most studies lacked data on self-reported body weight, as body weight is an objective “hard endpoint” that could have been used to internally validate the reported changes in diet and exercise behaviors.

Most importantly, while there were many studies covering pandemic-related changes in diet and physical activity in the general population, those included in this review that had age-specific data for older adults did not necessarily report age-specific results for all of the study outcomes [12, 14, 16, 17, 19-25, 27, 29, 31, 32, 37, 38]. This resulted in a relative dearth of information to work with despite the plethora of publications found in the initial literature search. We also note that more publications from low- and middle-income countries may have better illustrated the variation in changes globally.

However, despite these limitations, the results of our comprehensive synthesis of all relevant literature on the reported changes in diet and physical activity among older adults still justifies attention by health practitioners and public health officials, as the knowledge gained from this analysis can be used as an impetus to screen older populations for deleterious changes in lifestyle and intervene accordingly.

### Conclusion

Older individuals are a particularly vulnerable population who have been affected by the COVID-19 pandemic in several ways. Stay-at-home policies did not seem to have adversely affected their dietary habits but resulted in significant decreases in their physical activity. The extent of the effects of social distancing and self-isolation on the health of older individuals is just beginning to be understood. This review highlights the detrimental effects of COVID-19-related lockdowns, specifically for physical activity, and emphasizes the need for more focus on this age group. These results may guide public health organizations and primary care physicians to pay particular attention to how these changes have impacted the well-being of the older population and intervene as necessary.
